# Supplementary material for: Unveiling the Bioactive Potential of the Invasive Jellyfish Phyllorhiza punctata Through Integrative Transcriptomic and Proteomic Analyses
Source: Biomolecules. 2025 Aug 4;15(8):1121. doi: 10.3390/biom15081121 (PMC12383608; doi:10.3390/biom15081121)
Supplement: Supplementary file 1 [file biomolecules-15-01121-s001.zip › Suplementary Material/FileS3/PEPFOLD4/XP_065071592_15421/clusters.html]

 PEP-FOLD clusters


Download archive of all models  here   

|  |  |  |
| --- | --- | --- |
| # | file | sOPEP |
| # cluster 1 - Size: 2 |  |  |
|  | XP\_065071592\_15421-model1 | -25.0773 |
|  | XP\_065071592\_15421-model1.1 | -14.363 |
| # cluster 2 - Size: 7 |  |  |
|  | XP\_065071592\_15421-model2 | -19.0553 |
|  | XP\_065071592\_15421-model2.1 | -17.5188 |
|  | XP\_065071592\_15421-model2.2 | -17.2704 |
|  | XP\_065071592\_15421-model2.3 | -16.7578 |
|  | XP\_065071592\_15421-model2.4 | -16.2706 |
|  | XP\_065071592\_15421-model2.5 | -14.644 |
|  | XP\_065071592\_15421-model2.6 | -11.6626 |
| # cluster 3 - Size: 1 |  |  |
|  | XP\_065071592\_15421-model3 | -18.6 |
| # cluster 4 - Size: 7 |  |  |
|  | XP\_065071592\_15421-model4 | -17.7392 |
|  | XP\_065071592\_15421-model4.1 | -17.6122 |
|  | XP\_065071592\_15421-model4.2 | -16.7496 |
|  | XP\_065071592\_15421-model4.3 | -16.579 |
|  | XP\_065071592\_15421-model4.4 | -15.7444 |
|  | XP\_065071592\_15421-model4.5 | -15.1955 |
|  | XP\_065071592\_15421-model4.6 | -15.1189 |
| # cluster 5 - Size: 1 |  |  |
|  | XP\_065071592\_15421-model5 | -15.0503 |
| # cluster 6 - Size: 12 |  |  |
|  | XP\_065071592\_15421-model6 | -14.5532 |
|  | XP\_065071592\_15421-model6.1 | -13.0871 |
|  | XP\_065071592\_15421-model6.2 | -12.9005 |
|  | XP\_065071592\_15421-model6.3 | -12.5336 |
|  | XP\_065071592\_15421-model6.4 | -12.2632 |
|  | XP\_065071592\_15421-model6.5 | -12.1883 |
|  | XP\_065071592\_15421-model6.6 | -12.1687 |
|  | XP\_065071592\_15421-model6.7 | -11.7022 |
|  | XP\_065071592\_15421-model6.8 | -11.6376 |
|  | XP\_065071592\_15421-model6.9 | -11.6374 |
|  | XP\_065071592\_15421-model6.10 | -11.3991 |
|  | XP\_065071592\_15421-model6.11 | -11.1999 |
| # cluster 7 - Size: 1 |  |  |
|  | XP\_065071592\_15421-model7 | -14.429 |
| # cluster 8 - Size: 12 |  |  |
|  | XP\_065071592\_15421-model8 | -14.2936 |
|  | XP\_065071592\_15421-model8.1 | -14.042 |
|  | XP\_065071592\_15421-model8.2 | -12.358 |
|  | XP\_065071592\_15421-model8.3 | -12.2425 |
|  | XP\_065071592\_15421-model8.4 | -11.8747 |
|  | XP\_065071592\_15421-model8.5 | -11.8009 |
|  | XP\_065071592\_15421-model8.6 | -11.2455 |
|  | XP\_065071592\_15421-model8.7 | -11.0511 |
|  | XP\_065071592\_15421-model8.8 | -11.0331 |
|  | XP\_065071592\_15421-model8.9 | -10.9162 |
|  | XP\_065071592\_15421-model8.10 | -10.701 |
|  | XP\_065071592\_15421-model8.11 | -10.4732 |
| # cluster 9 - Size: 1 |  |  |
|  | XP\_065071592\_15421-model9 | -14.1817 |
| # cluster 10 - Size: 1 |  |  |
|  | XP\_065071592\_15421-model10 | -13.8928 |
| # cluster 11 - Size: 4 |  |  |
|  | XP\_065071592\_15421-model11 | -13.7031 |
|  | XP\_065071592\_15421-model11.1 | -13.5106 |
|  | XP\_065071592\_15421-model11.2 | -12.2184 |
|  | XP\_065071592\_15421-model11.3 | -11.0724 |
| # cluster 12 - Size: 1 |  |  |
|  | XP\_065071592\_15421-model12 | -13.1574 |
| # cluster 13 - Size: 2 |  |  |
|  | XP\_065071592\_15421-model13 | -13.1439 |
|  | XP\_065071592\_15421-model13.1 | -11.8183 |
| # cluster 14 - Size: 1 |  |  |
|  | XP\_065071592\_15421-model14 | -13.0465 |
| # cluster 15 - Size: 6 |  |  |
|  | XP\_065071592\_15421-model15 | -12.8997 |
|  | XP\_065071592\_15421-model15.1 | -12.7254 |
|  | XP\_065071592\_15421-model15.2 | -12.5985 |
|  | XP\_065071592\_15421-model15.3 | -12.2099 |
|  | XP\_065071592\_15421-model15.4 | -11.1119 |
|  | XP\_065071592\_15421-model15.5 | -10.8946 |
| # cluster 16 - Size: 1 |  |  |
|  | XP\_065071592\_15421-model16 | -12.5064 |
| # cluster 17 - Size: 1 |  |  |
|  | XP\_065071592\_15421-model17 | -12.4762 |
| # cluster 18 - Size: 1 |  |  |
|  | XP\_065071592\_15421-model18 | -12.4549 |
| # cluster 19 - Size: 2 |  |  |
|  | XP\_065071592\_15421-model19 | -12.4424 |
|  | XP\_065071592\_15421-model19.1 | -11.9524 |
| # cluster 20 - Size: 7 |  |  |
|  | XP\_065071592\_15421-model20 | -12.3699 |
|  | XP\_065071592\_15421-model20.1 | -12.1723 |
|  | XP\_065071592\_15421-model20.2 | -12.0159 |
|  | XP\_065071592\_15421-model20.3 | -11.6716 |
|  | XP\_065071592\_15421-model20.4 | -11.5548 |
|  | XP\_065071592\_15421-model20.5 | -11.4498 |
|  | XP\_065071592\_15421-model20.6 | -11.1354 |
| # cluster 21 - Size: 2 |  |  |
|  | XP\_065071592\_15421-model21 | -12.2822 |
|  | XP\_065071592\_15421-model21.1 | -10.8395 |
| # cluster 22 - Size: 10 |  |  |
|  | XP\_065071592\_15421-model22 | -12.2672 |
|  | XP\_065071592\_15421-model22.1 | -11.9202 |
|  | XP\_065071592\_15421-model22.2 | -11.7371 |
|  | XP\_065071592\_15421-model22.3 | -11.6414 |
|  | XP\_065071592\_15421-model22.4 | -11.5072 |
|  | XP\_065071592\_15421-model22.5 | -11.4811 |
|  | XP\_065071592\_15421-model22.6 | -11.2226 |
|  | XP\_065071592\_15421-model22.7 | -11.1143 |
|  | XP\_065071592\_15421-model22.8 | -11.0359 |
|  | XP\_065071592\_15421-model22.9 | -10.8076 |
| # cluster 23 - Size: 3 |  |  |
|  | XP\_065071592\_15421-model23 | -12.0984 |
|  | XP\_065071592\_15421-model23.1 | -11.8734 |
|  | XP\_065071592\_15421-model23.2 | -11.3508 |
| # cluster 24 - Size: 3 |  |  |
|  | XP\_065071592\_15421-model24 | -11.9002 |
|  | XP\_065071592\_15421-model24.1 | -11.7276 |
|  | XP\_065071592\_15421-model24.2 | -11.0469 |
| # cluster 25 - Size: 6 |  |  |
|  | XP\_065071592\_15421-model25 | -11.753 |
|  | XP\_065071592\_15421-model25.1 | -11.7283 |
|  | XP\_065071592\_15421-model25.2 | -11.5434 |
|  | XP\_065071592\_15421-model25.3 | -11.5208 |
|  | XP\_065071592\_15421-model25.4 | -11.4838 |
|  | XP\_065071592\_15421-model25.5 | -10.9106 |
| # cluster 26 - Size: 1 |  |  |
|  | XP\_065071592\_15421-model26 | -11.5038 |
| # cluster 27 - Size: 1 |  |  |
|  | XP\_065071592\_15421-model27 | -11.4862 |
| # cluster 28 - Size: 1 |  |  |
|  | XP\_065071592\_15421-model28 | -11.3623 |
| # cluster 29 - Size: 1 |  |  |
|  | XP\_065071592\_15421-model29 | -11.0503 |
| # cluster 30 - Size: 1 |  |  |
|  | XP\_065071592\_15421-model30 | -10.5054 |
|  |  |  |
